# Die medizinische Habilitation an deutschen Hochschulen: ein Vergleich der Ordnungen über 23 Jahre

**DOI:** 10.1007/s00104-021-01545-z

**Published:** 2021-12-14

**Authors:** H. Sorg, J. Ehlers, M. Bagheri, P. C. Fuchs, C. G. G. Sorg

**Affiliations:** 1 Lehrstuhl für Didaktik und Bildungsforschung im Gesundheitswesen, Fakultät für Gesundheit, Universität Witten-Herdecke, Alfred-Herrhausen-Str. 50, 58448 Witten, Deutschland; 2 Klinik für Plastische, Rekonstruktive, Ästhetische und Handchirurgie, Klinikum Westfalen, Dortmund, Deutschland; 3 Klinik für Plastische Chirurgie, Handchirurgie, Schwerbrandverletztenzentrum, Universität Witten/Herdecke, Witten/Herdecke, Deutschland; 4 Klinikum Köln Merheim, Köln, Deutschland; 5 Lehrstuhl für Management und Innovation im Gesundheitswesen, Fakultät für Wirtschaft und Gesellschaft, Universität Witten/Herdecke, Witten, Deutschland

**Keywords:** Habilitationsordnung, Akademische Qualifikation, Habilitationsscore, Voraussetzungen, Langzeitvergleich, Habilitation regulation, Academic qualification, Habilitation score, Prerequisites, Long-term comparison

## Abstract

**Hintergrund:**

Aufgrund der seit 2010 weiter gesunkenen Anzahl an Habilitationen in der Medizin könnten die generellen Anforderungen an die Habilitation im gleichen Zeitraum gestiegen sein.

**Ziel der Arbeit:**

Die Anforderungen für eine medizinische Habilitation an deutschen Hochschulen im Vergleich von 23 Jahren werden reevaluiert.

**Material und Methoden:**

Es erfolgte eine Analyse der Habilitationsordnungen auf 12 Zielparameter und Bewertung dieser durch ein Scoringsystem (Range 0–34 Punkte).

**Ergebnisse:**

Lediglich das Kriterium der Promotionsvoraussetzung ist im 23-Jahres-Vergleich in der Bewertung gleichgeblieben (1998–2021). Alle Ergebnisse der anderen 11 Kriterien haben sich im Vergleich zur Voruntersuchung aus 2010 verändert. Die Bewertung der Habilitationsleistungen ist von einem Gesamtscore aus dem Jahr 1998 von 15,2 ± 5,1 (95 %-Konfidenzintervall 13,6–16,9) auf nun 25,1 ± 3,6 Punkte im Jahr 2021 gestiegen (95 %-Konfidenzintervall 23,9–26,2; *p* < 0,001). Die Range der vergebenen Scoringwerte ist im 11-Jahres-Vergleich wieder breiter gestreut mit Werten von 12 bis 31 Punkten. Als auffällig neues Kriterium zeigte sich, dass bei 98 % der bewerteten Habilitationsordnungen nun eine didaktische Weiterbildung in jedoch erheblich unterschiedlicher Anforderung von den Fakultäten gefordert wird.

**Schlussfolgerung:**

Die Anforderungen an eine medizinische Habilitation sind im 23-Jahres-Vergleich weiter signifikant angestiegen mit jedoch breiterer Streuung der Scorings. Die detailliertere Beschreibung kann als direkter Hinweis auf eine Verbesserung der Transparenz angesehen werden. Die breitere Streuung zeigt hingegen, dass ein einheitlicher Bewertungsmaßstab für Deutschland wieder in die Ferne gerückt ist.

## Abstract (en)

**Background:**

Due to the further decrease in the number of habilitations in medicine since 2010, the general requirements for habilitation could have increased during the same period.

**Objective:**

The requirements for a medical habilitation at German universities in a comparison of 23 years are re-evaluated.

**Material and methods:**

An analysis of habilitation regulations for 12 target parameters and evaluation of these by a scoring system (range 0–34 points).

**Results:**

Only the criterion of the requirement for a doctorate has remained the same in the 23-year comparison in the evaluation (1998–2021). All results of the other 11 criteria have changed compared to the previous study from 2010. The rating of habilitation achievements has increased from a total score in 1998 of 15.2 ± 5.1 points (95% confidence interval 13.6–16.9 points) to 25.1 ± 3.6 points in 2021 (95% confidence interval 23.9–26.2 points; *p* < 0.001). The range of assigned scoring values is again more broadly spread in the 11-year comparison with values from 12 to 31 points. A striking new criterion was that 98% of the assessed habilitation regulations now require a didactic continuing education in, however, significantly different requirements from the faculties.

**Conclusion:**

The requirements for a medical habilitation continued to significantly increase over the 23-year period with, however, a wider dispersion of scores. The more detailed description can be seen as a direct indication of an improvement in transparency. In contrast, the broader dispersion shows that a uniform assessment standard for Germany has again receded into the distance.

## Hintergrund

Die Habilitation in der Medizin ist und bleibt eine wichtige akademische Prüfung, welche trotz viel Kritik und Diskussion der letzten Jahre weiterhin einen bedeutenden Stellenwert für die Universitäten und Hochschulen wie auch die Karriere einer Ärztin oder eines Arztes hat [2, 7, 11, 12]. Zwar haben die Zahlen für abgeschlossene Habilitationen im Fachgebiet der Humanmedizin und den Gesundheitswissenschaften seit 2010 um 8,1 % abgenommen [9], bleiben im Fachgruppenvergleich hingegen weiterhin für mehr als die Hälfte der Habilitationen in Deutschland verantwortlich [9]. Erfreulicherweise ist bei diesem Negativtrend allerdings, dass im gleichen Zeitraum die Anzahl an Habilitandinnen um 26,3 % gestiegen ist [9]. Frauen habilitieren jedoch deutlich häufiger in konservativ als in operativ tätigen Fachgebieten [3]. So habilitieren z. B. im Fachgebiet der Neurologie alleine 29 % der Frauen, während dies im Gebiet der Chirurgie nur 14 % tun [3].

In der Untersuchung der Habilitationsordnungen von 2010 konnte bereits eine Vereinheitlichung der Anforderungen an die medizinische Habilitation in Deutschland festgestellt werden [5]. Neben dem Ruf nach mehr Transparenz und weniger Abhängigkeit von Ordinarien im Habilitationsverfahren wurde in der Folge die Vereinheitlichung der deutschlandweiten Habilitationsanforderungen von bereits Habilitierten als Hauptreformwunsch genannt [7]. In einer quantitativen Studie zum Inhalt und zur Ausgestaltung der Habilitationsrichtlinien im deutschsprachigen Raum aus dem Jahr 2013 unter Verwendung von 87 Indikatoren konnte keine Vereinheitlichung festgestellt werden [13]. Im Gegenteil wurden in dieser Studie teilweise erhebliche Unterschiede in den Qualifikations- und Verfahrenskriterien in den meisten Habilitationsordnungen abgeleitet [13]. Unter Nutzung anderer Parameter war auch in einer weiteren Untersuchung aus dem Jahr 2020 keine wesentliche Vereinheitlichung der Habilitationsordnungen im Fachgebiet der Medizin ersichtlich [10]. Im gleichen Kanon wurde auch beim Teilschritt der Verleihung der Venia Legendi eine Beseitigung der teilweise erheblichen Unterschiede in den Anforderungen jeweiliger Hochschulen gefordert, um der Chancengleichheit in Deutschland gerecht zu werden [2].

In Anbetracht der über einen 12-Jahres-Zeitraum (1998–2010) gestiegenen Anforderungen an eine medizinische Habilitation in Deutschland und in der Folge dem Rückgang der Habilitation in der Medizin stellte sich nun die Frage, ob der Rückgang im Folgezeitraum (2010–2021) durch weitere Steigerungen der Habilitationsbedingungen begründet sein könnte.

## Methoden

Die Untersuchungen wurden im Rahmen einer Langzeitanalyse des Studienarms I der Karriere-in-der-Medizin(KARiMED)-Studie (www.karrierestudie.de) durchgeführt.

### Recherche der Habilitationsordnungen.

Die Habilitationsordnungen und jeweiligen Ausführungsbestimmungen der medizinischen Fakultäten oder Hochschulen in Deutschland wurden von der relevanten Internetseite heruntergeladen oder per E‑Mail von der jeweiligen Fakultät angefordert. Konnten entsprechende Informationen nicht aus den Vorschriften oder deren Begleitunterlagen gefunden werden, wurden die Einrichtungen zusätzlich per E‑Mail oder Telefon kontaktiert, um fehlende Informationen zu erhalten. Insgesamt 37 von 40 (92,5 %) Habilitationsordnungen konnten auf diese Weise in die Analyse eingeschlossen werden. Bei drei Fakultäten war es dem Autorenteam nicht möglich, an die gültige Habilitationsordnung zu gelangen, da die Fakultäten noch im Aufbau befindlich sind und aktuell an ihren Ordnungen arbeiten.

### Zielkriterien.

Zielparameter waren die Habilitationsvoraussetzungen, welche bereits 1998 durch Nagelschmidt et al. [6] beschrieben und 2010 durch Knobloch et al. [5] verwendet wurden. Dieser Score und der Bewertungsmaßstab sind erneut die Basis dieser vorliegenden Untersuchung.

### Einschlusskriterien.

Einschlusskriterium war das Vorliegen einer Habilitationsordnung einer deutschen medizinischen Fakultät.

### Ausschlusskriterien.

Ausschlusskriterium war: keine dokumentierten Angaben über die Habilitationsordnung einer deutschen medizinischen Fakultät.

Aus den Habilitationsordnungen und den beiliegenden Ergänzungen und Merkblättern wurden die Vorschriften auf detaillierte Angaben zu den oben genannten Kriterien, insbesondere im Hinblick auf die Qualität der Habilitationsleistungen von zwei unabhängigen Bewertern untersucht und bewertet. Die entsprechenden Angaben wurden in Gruppen mit ähnlichem Anforderungsprofil zusammengefasst und mit einem einfachen Punktesystem nach zunehmender Präzisierung und steigender Anforderung bewertet. Dabei wurden die von allen öffentlichen Hochschulen geforderten Leistungsnachweise mit maximal 5 Punkten bewertet, die nicht einheitlich geforderten mit geringeren Punktzahlen. Insofern besteht die Möglichkeit, Punktwerte zwischen 0 und 34 für die Habilitationsordnung einer Universität zu erzielen. Die einzelnen Habilitationsvoraussetzungen werden nach den zwölf Kriterien wie folgt bewertet:

1. Promotion
  0 Punkte: keine Angaben
  1 Punkt: Promotion muss vorliegen
2. Wissenschaftliche Publikation
  0 Punkte: keine näheren Angaben
  1 Punkt: diffuser Hinweis auf die notwendige Anzahl ± hinreichende Anzahl
  2 Punkte: Hinweise auf die Qualität
    ± z. B. in einschlägigen wissenschaftlichen Fachzeitschriften publiziert, in Current Contents gelistet
  3 Punkte: Hinweise auf Autorenschaften und Qualität
    ± z. B. möglichst Alleinautorenschaft, Arbeiten mit Kreativität, die wissenschaftliche Befähigung nachweisen
  4 Punkte: Hinweise auf Anzahl und Qualität
    ± z. B. ausreichende/angemessene Anzahl in international renommierten Fachzeitschriften
    6, 10 oder 12 Originalpublikationen in international anerkannten Fachzeitschriften
  5 Punkte: Hinweise auf Anzahl, Qualität und Autorenschaften
    10 Publikationen, davon 6‑mal Erstautor, in Fachzeitschriften mit Peer-review-Verfahren
    8 bis 10 hochrangige Originalpublikationen, überwiegend Erstautor
    10 Publikationen als Erstautor, davon die Hälfte in renommierten Fachzeitschriften mit Gutachterverfahren
    12 Originalpublikationen, 8‑mal Erstautor, Impact-Faktor 10 in renommierten Fachzeitschriften mit Gutachterverfahren
3. Probevortrag
  0 Punkte: keine näheren Angaben
  1 Punkt: keine genaue Beschreibung des Vortrags, keine Themenauswahl
  2 Punkte: es müssen für den Probevortrag 3 Themen zur Auswahl vorgelegt werden
  3 Punkte: Vorlage von 3 Themen, welche nicht das Habilitationsthema betreffen
  4 Punkte: Disputation über die Habilitationsarbeit
  5 Punkte: 2 Veranstaltungen notwendig: Probevortrag mit Themenauswahl sowie Vortrag über die Habilitationsarbeit mit anschließender Disputation
4. Schriftliche Habilitationsleistung
  0 Punkte: keine näheren Angaben zur Qualität der Habilitationsarbeit
  1 Punkt: selbständig verfasste Leistung
  2 Punkte: Zusammenfassung wissenschaftlich wertvoller Ergebnisse, wesentlicher Erkenntnisse oder Ergebnisse mit hohem Niveau
  3 Punkte: Erkennbarkeit der Eignung zur Professorin oder zum Professor
  4 Punkte: Kombination der o. g. Forderungen
  5 Punkte: zusätzliche Qualitätsmerkmale
5. Lehrtätigkeit
  0 Punkte: keine Angaben bzw. fakultativ (Nachweis falls vorhanden)
  1 Punkt: Nachweis ohne Vorgaben (z. B. Verzeichnis vorlegen, Übersicht über die Lehrtätigkeit)
  2 Punkte: mit Qualitätsforderung (in der Lehre erfolgreich mitgewirkt)
  3 Punkte: mit quantitativer Forderung, mindestens 1 Semester (z. B. Nachweis einer einsemestrigen studiengangbezogenen Lehrveranstaltung von mindestens 15 h)
  4 Punkte: mit quantitativer Forderung, mehrsemestrige Tätigkeit (z. B. mindestens 4 Semester bei Pflichtveranstaltungen, 4 Jahre praktische Tätigkeit in der Lehre)
6. Anerkennung als Fachärztin/Facharzt/Weiterbildung
  0 Punkte: keine Angaben
  1 Punkt: nach der Promotion mehrere Jahre im Fach
  2 Punkte: Fachärztin/Facharzt oder vergleichbare Ausbildung in anderen Fächern
  3 Punkte: Fachärztin/Facharzt und/oder 4 Jahre im Fach tätig
7. Wissenschaftliche Vorträge/Poster
  0 Punkte: keine Angaben über gehaltene wissenschaftliche Vorträge oder Poster
  1 Punkt: Verzeichnis der gehaltenen wissenschaftlichen Vorträge oder Poster
  2 Punkte: genaue Auflistung der wissenschaftlichen Vorträge oder Poster mit Angaben zu Anzahl, Qualität und Autorenschaft oder Angabe der 5 wichtigsten/bedeutendsten Vorträge
8. Antrittsvorlesung
  0 Punkte: keine Angabe
  1 Punkt: Abhaltung einer Antrittsvorlesung nach erfolgreichem Abschluss des Habilitationsverfahrens
9. Nachweis der Lehrbefähigung
  0 Punkte: keine Angaben
  1 Punkt: Lehrbefähigung erwähnt ohne spezielle Forderung (z. B. wird durch den Probevortrag beurteilt, Kommission informiert sich)
  2 Punkte: Nachweis durch Lehrveranstaltung, Überprüfung fakultativ (z. B. Eignung aufgrund einer studiengangbezogenen Lehrveranstaltung nachweisen, spezielle Lehrveranstaltung kann von der Dekanin oder dem Dekan gefordert werden)
  3 Punkte: Probevorlesung zur Überprüfung der didaktischen Eignung mit Votum durch Studierende oder Fakultät
10. Wissenschaftliche Tätigkeit (WT)
  0 Punkte: keine Angaben
  1 Punkt: nach Promotion wissenschaftlich tätig gewesen oder WT an wissenschaftlicher Hochschule
  2 Punkte: qualifizierte bzw. über die Promotion hinausgehende WT oder mehrjährige (erfolgreiche) WT
  3 Punkte: mindestens 3 Jahre im Fachgebiet in der Forschung tätig gewesen oder mindestens 4 Jahre im Habilitationsfach oder 5 Jahre in wissenschaftlichen Instituten/Einrichtungen tätig gewesen
11. Vertraut mit der Breite des Faches
  0 Punkte: keine näheren Angaben zur Vertrautheit im angestrebten Habilitationsfach
  1 Punkt: vertraut mit der Breite des Fachs bzw. Kenntnisse anderer Fachprobleme
12. Vorstellung vor der Fakultät
  0 Punkte: keine Vorstellung notwendig
  1 Punkt: Vorstellung vor der Fakultät vor Antragstellung wird erwartet

## Ergebnisse

### Gesamtscore

**Figure 1:**
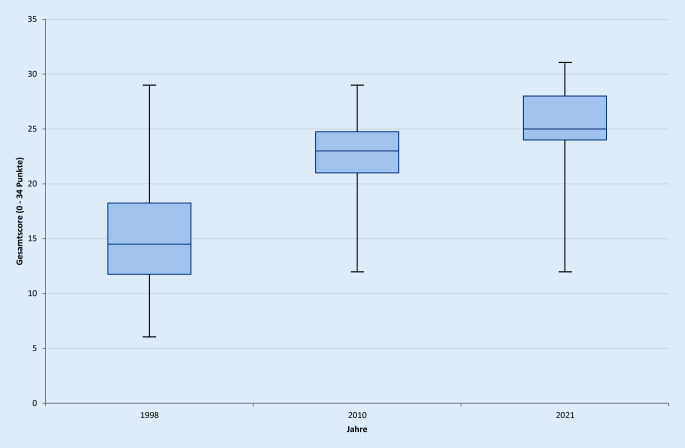

Der Gesamtscore für die Habilitationsleistung in Medizin ist in einem 23-Jahres-Vergleich von 15,2 ± 5,1 (95 %-Konfidenzintervall 13,6–16,9) im Jahre 1998 auf 21,9 ± 4,0 Punkte (95 %-Konfidenzintervall 20,6–23,3; *p* < 0,001) im Jahr 2010 auf nun 25,1 ± 3,6 (95 %-Konfidenzintervall 23,9–26,2; *p* < 0,001) gestiegen (Abb. 1 und 2).

**Figure 2:**
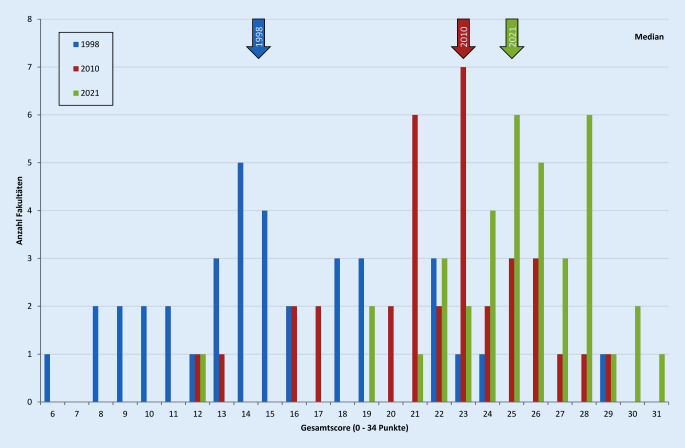

### Alter der Habilitationsordnungen

Die Habilitationsordnungen sind in der aktuellen Bewertung etwas älter geworden mit 7,1 ± 1,0 Jahre (1998: 7,7 ± 1,0 Jahre; 2010: 6,0 ± 1,0 Jahre), wobei die älteste 25 Jahre alt war und die aktuellsten (*n* = 2) aus dem Jahr 2020 stammten [5, 6]. Hierbei muss berücksichtigt werden, dass die zu den Ordnungen bestehenden Ausführungsbestimmungen teilweise jünger sind. Dies ermöglicht es den jeweiligen Fakultäten, die Voraussetzungen schneller anzupassen. Führt jedoch auf der anderen Seite mitunter zu unterschiedlichen Anforderungen an den Lehrkörper innerhalb der Universität zwischen den unterschiedlichen Fachgebieten.

### Habilitationsvoraussetzungen

Generell lässt sich feststellen, dass sich insgesamt 11 der 12 bewerteten Kriterien im Vergleich zu 2010 verändert haben. Hier wurden insgesamt 4 der Kriterien höher bewertet, 5 geringer und 3 sind im 11-Jahres-Zeitraum gleichgeblieben. Hierbei muss allerdings berücksichtigt werden, dass bei der hier dargestellten Untersuchung 3 Fakultäten mehr in die Untersuchung eingeschlossen werden konnten (*n* = 37) als im Jahre 2010 (*n* = 34) und eine mehr als 1998 (*n* = 36; Tab. 1). Grundsätzlich zeigt sich auch eine breitere Streuung der Scoringwerte von 12 bis 31 Punkten (Abb. 2).

**Table 1:** 

	1998 (*n* = 36)			2010 (*n* = 34)			2021 (*n* = 37)	
Anforderung	(*n*)	(%)		(*n*)	(%)		(*n*)	(%)
1. Promotion	36	100,0	=	34	100,0	=	37	100,0
2. Wissenschaftliche Publikation	36	100,0	=	34	100,0	↓	35	94,6
3. Probevortrag	36	100,0	↓	32	94,1	↑	36	97,3
4. Schriftliche Habilitationsleistung	35	97,2	↑	34	100,0	=	37	100,0
5. Lehrtätigkeit	31	86,1	↑	34	100,0	=	37	100,0
6. Anerkennung als Fachärztin/Facharzt	24	66,7	↑	30	88,2	↓	32	86,5
7. Wissenschaftliche Vorträge/Poster	24	66,7	↓	22	64,7	↑	27	73,0
8. Antrittsvorlesung	22	61,1	↓	18	52,9	↑	30	81,1
9. Nachweis der Lehrbefähigung	18	50,0	↑	34	100,0	↓	35	94,6
10. Wissenschaftliche Tätigkeit	17	47,2	↑	34	100,0	↓	32	86,5
11. Vertraut mit der Breite des Faches	10	27,8	↑	11	32,4	↓	11	29,7
12. Vorstellung vor der Fakultät	7	19,4	↓	2	5,9	↑	9	24,3

*n* Anzahl der Fakultäten; ↑ gestiegene Anforderung im Jahresvergleich; ↓ gesunkene Anforderung im Jahresvergleich; = gleichgebliebene Anforderung im Jahresvergleich

#### Promotion (1)

Als einziges Kriterium ist die Promotionsvoraussetzung über 23 Jahre unverändert geblieben und gilt an allen eingeschlossenen Fakultäten als Voraussetzung für eine Habilitation. Ergänzend haben wir die Ordnungen nach einer notwendigen Mindestanforderung an die Zugangsvoraussetzung der Promotion untersucht. Sechs der 37 (16,2 %) Fakultäten fordern mittlerweile eine Promotion mit herausragender Beurteilung (magna cum laude).

#### Wissenschaftliche Publikation (2)

In 94,6 % der untersuchten Habilitationsordnungen wird die publikatorische Leistung detailliert beschrieben. Hierfür erhalten 34 Fakultäten (91,9 %) die höchste Punktbewertung (5 Punkte) – im Vergleich zu 2010 ein klarer Trend zu höherer Anforderung bzw. detaillierter Beschreibung. Zwei Fakultäten machen in der neuen Bewertung hierzu jedoch keine Angaben (Tab. 2).

**Table 2:** 

		1998 (*n* = 36)			2010 (*n* = 34)			2021 (*n* = 37)	
Punkte	Angabe	(*n*)	(%)		(*n*)	(%)		(*n*)	(%)
0	Keine näheren Angaben	18	50,0	↓	0	0,0	↑	2	5,4
1	Diffuser Hinweis auf die notwendige Anzahl hinreichende Anzahl	1	2,8	↑	5	14,7	↓	0	0,0
2	Hinweise auf die Qualität z. B. in einschlägigen wissenschaftlichen Fachzeitschriften publiziert, in Current Contents gelistet	3	8,3	↓	2	5,9	↓	0	0,0
3	Hinweise auf Autorenschaften und Qualität z. B. möglichst Alleinautorenschaft, Arbeiten mit Kreativität, die wissenschaftliche Befähigung nachweisen	2	5,6	↓	1	2,9	↓	1	2,7
4	Hinweise auf Anzahl und Qualität z. B. ausreichende/angemessene Anzahl in international renommierten Fachzeitschriften 6, 10 oder 12 Originalpublikationen in international anerkannten Fachzeitschriften	8	22,2	↓	6	17,6	↓	0	0,0
5	Hinweise auf Anzahl, Qualität und Autorenschaften 10 Publikationen, davon 6‑mal Erstautor, in Fachzeitschriften mit Peer-review-Verfahren 8 bis 10 hochrangige Originalpublikationen, überwiegend Erstautor 10 Publikationen als Erstautor, davon die Hälfte in renommierten Fachzeitschriften mit Gutachterverfahren 12 Originalpublikationen, 8‑mal Erstautor, Impact-Faktor, 10 in renommierten Fachzeitschriften mit Gutachterverfahren	4	11,1	↑	20	58,8	↑	34	91,9

*n* = Anzahl der Fakultäten; ↑ gestiegene Anforderung im Jahresvergleich; ↓ gesunkene Anforderung im Jahresvergleich

#### Probevortrag (3)

Generell kam es im 11-Jahres-Vergleich zu einem Downgrading der Bewertungen mit einer höheren Anzahl an Fakultäten mit niedrigeren Scoringbewertungen. Zum Probevortrag macht nur eine Fakultät keine näheren Angaben (Score 0) und 4 Fakultäten beschreiben zwar die Notwendigkeit, jedoch ohne nähere Beschreibung oder etwaige Themenauswahl (Score 1). Der Score mit 2 Punkten wurde nicht vergeben. In den anderen Fällen sind Probevorträge unmittelbare Voraussetzung (Score 3: *n* = 19; Score 4: *n* = 8; Score 5: *n* = 4).

#### Schriftliche Habilitationsleistung (4)

In allen eingeschlossenen Habilitationsordnungen wird sowohl die Monographie als auch die kumulative Habilitationsschrift gefordert und ermöglicht. Auch zur Ausgestaltung machen alle Ordnungen bzw. Ausführungsbestimmungen nun Angaben. So soll die Habilitationsschrift an 6 Fakultäten eine selbstständig verfasste Leistung sein (Score 1) an 9 Fakultäten eine Zusammenfassung wissenschaftlich wertvoller oder wesentlicher Erkenntnisse bzw. Ergebnisse (Score 2) und an 7 Orten soll die Eignung zur Professorin oder zum Professor erkennbar sein, jedoch in Kombination zu den vorher genannten Voraussetzungen (Score 4). An 15 Fakultäten muss die Habilitationsschrift zusätzliche Qualitätsmerkmale beinhalten. Die Anzahl der notwendigen Publikationen und auch die Qualitätsvoraussetzungen (z. B. gewünschte Impact-Faktoren) für eine kumulative Habilitationsschrift variieren weiter teilweise erheblich zwischen den Fakultäten. Die Möglichkeit der Anerkennung von Dissertationsarbeiten als Habilitationsschrift ist an keiner Fakultät mehr möglich.

#### Lehrtätigkeit (5)

Die Lehrtätigkeit wird seit 2010 in den Ordnungen detaillierter beschrieben. So erreichen 29 Habilitationsordnungen einen Punktwert von 4 im Jahr 2021 (Tab. 3). Weiterhin können viele Arten der Lehre (Vorlesungen, Seminare, Praktika u. a.) als Lehrleistung angerechnet werden. In den Ausführungsbestimmungen werden diese zumeist transparent und nachvollziehbar erläutert. Die zeitlichen Mindestanforderungen im Sinne geforderter Semesterwochenstunden variieren weiterhin erheblich.

**Table 3:** 

		1998 (*n* = 36)			2010 (*n* = 34)			2021 (*n* = 37)	
Punkte	Angabe	(*n*)	(%)		(*n*)	(%)		(*n*)	(%)
0	Keine Angaben bzw. fakultativ Nachweis falls vorhanden	5	13,9	↓	0	0,0	=	0	0,0
1	Nachweis ohne Vorgaben z. B. Verzeichnis vorlegen Übersicht über die Lehrtätigkeit	15	41,7	↓	10	29,4	↓	5	13,5
2	Mit Qualitätsforderung in der Lehre erfolgreich mitgewirkt	2	5,6	↑	2	5,9	↓	2	5,4
3	Mit quantitativer Forderung, mindestens 1 Semester z. B. Nachweis einer einsemestrigen studiengangbezogenen Lehrveranstaltung von mindestens 15 h	5	13,9	↓	3	8,8	↓	1	2,7
4	Mit quantitativer Forderung, mehrsemestrige Tätigkeit z. B. mindestens 4 Semester bei Pflichtveranstaltungen 4 Jahre praktische Tätigkeit in der Lehre	9	25,0	↑	19	55,9	↑	29	78,4

*n* Anzahl der Fakultäten; ↑ gestiegene Anforderung im Jahresvergleich; ↓ gesunkene Anforderung im Jahresvergleich; = gleichgebliebene Anforderung im Jahresvergleich

#### Anerkennung als Fachärztin/Facharzt/Weiterbildung (6)

Die Weiterbildung zur Fachärztin bzw. zum Facharzt (sofern möglich) wird an 32 von 37 Fakultäten vorausgesetzt (Tab. 4). In diesem Zusammenhang fordern nun 30 Fakultäten explizit die Anerkennung oder eine vergleichbare Ausbildung (Score 2: *n* = 30) bzw. die abgeschlossene Facharztweiterbildung und/oder 4 Jahre Tätigkeit im Fachgebiet (Score 3: *n* = 2). Fünf Fakultäten machen keine weiteren Angaben. Interessanterweise wird in 4 Habilitationsordnungen den Kandidatinnen und Kandidaten ohne (bisherige) Facharztanerkennung, auch die Möglichkeit geboten, mit dem Zusatz „experimentelle“ in einem klinischen Fachgebiet zu habilitieren. Dies war aus den bisherigen Untersuchungen so nicht bekannt bzw. wurde nicht in den Ordnungen verschriftlicht.

**Table 4:** 

		1998 (*n* = 36)			2010 (*n* = 34)			2021 (*n* = 37)	
Punkte	Angabe	(*n*)	(%)		(*n*)	(%)		(*n*)	(%)
0	Keine Angaben	10	27,8	↓	4	11,8	↑	5	13,5
1	Nach der Promotion mehrere Jahre im Fach	2	5,6	↓	1	2,9	↓	0	0,0
2	Fachärztin/Facharzt oder vergleichbare Ausbildung in anderen Fächern	17	47,2	↑	23	67,6	↑	30	81,1
3	Fachärztin/Facharzt und/oder 4 Jahre im Fach tätig	7	19,4	↓	6	17,6	↓	2	5,4

*n* Anzahl der Fakultäten; ↑ gestiegene Anforderung im Jahresvergleich; ↓ gesunkene Anforderung im Jahresvergleich

#### Wissenschaftliche Vorträge/Poster (7)

Die Präsentation wissenschaftlicher Daten ist, auch gerade für die Sichtbarkeit geleisteter Forschungstätigkeit neben der Publikation, von großer Bedeutung. Waren es 1998 nur 33 % der Fakultäten, welche wissenschaftlichen Vorträgen und Postern keinerlei Bedeutung zuteilten, so ist der Anteil 2021 auf 10 Fakultäten (27 %) geschrumpft. Lediglich 3 Fakultäten haben indes den Weg beschritten, Mindestvorgaben zu machen, in welchem eine verbindliche Anzahl an wissenschaftlichen Vorträgen und Postern festgeschrieben wurde (z. B. 12 Vorträge).

#### Antrittsvorlesung (8)

An 81 % der medizinischen Fakultäten in Deutschland ist eine Antrittsvorlesung notwendig, was im Vergleich zu 2010 einem Plus von 28 % der untersuchten Fakultäten entspricht.

#### Nachweis der Lehrbefähigung (9)

Die Einzelleistungen zur Lehrbefähigung bleiben insgesamt heterogen, während die Bewertung der Lehrbefähigung eine Tendenz hin zu einer genaueren Beschreibung mit höheren Scoringwerten im 23-Jahres-Vergleich hat. Wurde dieser Nachweis im Jahr 2010 noch von allen Ordnungen verlangt, wird in der aktuellen Bewertung an 2 Fakultäten hierzu keine Angabe gemacht. Jedoch kommt es zu einer Steigerung der Bewertung mit 3 Scoringpunkten, welche vergeben wurden, wenn eine Probevorlesung zur Überprüfung der didaktischen Eignung mit Votum durch Studierende oder Fakultät notwendig wird (Tab. 5).

**Table 5:** 

		1998 (*n* = 36)			2010 (*n* = 34)			202 (*n* = 37)	
Punkte	Angabe	(*n*)	(%)		(*n*)	(%)		(*n*)	(%)
0	Keine Angaben	12	33,3	↓	0	0,0	↑	2	5,4
1	Lehrbefähigung erwähnt ohne spezielle Forderung z. B. wird durch den Probevortrag beurteilt Kommission informiert sich	4	11,1	↑	4	11,8	↓	2	5,4
2	Nachweis durch Lehrveranstaltung, Überprüfung fakultativ z. B. Eignung aufgrund einer studiengangbezogenen Lehrveranstaltung nachweisen spezielle Lehrveranstaltung kann vom Dekan gefordert werden	6	16,7	↓	5	14,7	↓	5	13,5
3	Probevorlesung zur Überprüfung der didaktischen Eignung mit Votum durch Studenten oder Fakultät	14	38,9	↑	25	73,5	↑	28	75,7

*n* Anzahl der Fakultäten; ↑ gestiegene Anforderung im Jahresvergleich; ↓ gesunkene Anforderung im Jahresvergleich

Wurde im Jahr 2010 noch von 20 Fakultäten eine Fortbildung in Didaktik gefordert, so hat diese Habilitationsvoraussetzung nun bei 35 Fakultäten Einzug gehalten (Steigerung um 75 %). Die Spanne der hierfür geforderten Stundenzahl reicht von 8 bis 200 h (44,8 h Mittelwert) und setzt teilweise den erfolgreichen Abschluss eines Didaktikseminars mit Zertifikat voraus.

#### Wissenschaftliche Tätigkeit (10)

Im Jahr 1998 wurden bei 53 % der Fakultäten keine definitiven Forderungen zur wissenschaftlichen Tätigkeit gemacht. Im Jahr 2010 war dies dann in allen Ordnungen (100 %) verankert. Für die aktuelle Bewertung werden an 5 Standorten hierzu wiederum keine Angaben mehr gemacht. In der folgenden Scoringbewertung zeigt sich, dass es an 6 Fakultäten ausreicht, nach der Promotion wissenschaftlich tätig oder an einer wissenschaftlichen Einrichtung gewesen zu sein. Fünfzehn Habilitationsordnungen machen nähere Angaben im Sinne einer mehrjährigen qualifizierten über die Promotion hinausgehenden wissenschaftlichen Tätigkeit (entsprechend Score 2). Eine 3‑ bis 5‑jährige wissenschaftliche Tätigkeit im Fachgebiet bzw. im Habilitationsfach findet sich in 11 der 37 Habilitationsordnungen wieder (Tab. 6).

**Table 6:** 

		1998 (*n* = 36)			2010 (*n* = 34)			2021 (*n* = 37)	
Punkte	Angabe	(*n*)	(%)		(*n*)	(%)		(*n*)	(%)
0	Keine Angaben	19	52,8	↓	0	0,0	↑	5	13,5
1	Nach Promotion wissenschaftlich tätig gewesen oder WT an wissenschaftlicher Hochschule	3	8,3	↑	8	23,5	↓	6	16,2
2	Qualifizierte bzw. über die Promotion hinausgehende WT oder mehrjährige (erfolgreiche) WT	5	13,9	↑	16	47,1	↓	15	40,5
3	Mindestens 3 Jahre im Fachgebiet in der Forschung tätig gewesen oder mindestens 4 Jahre im Habilitationsfach oder 5 Jahre in wissenschaftlichen Instituten/Einrichtungen tätig gewesen	9	25,0	↑	10	29,4	↑	11	29,7

*n* Anzahl der Fakultäten; *WT* wissenschaftliche Tätigkeit; ↑ gestiegene Anforderung im Jahresvergleich; ↓ gesunkene Anforderung im Jahresvergleich

#### Vertraut mit der Breite des Faches (11)

Nagelschmidt und Kollegen haben bereits vor 23 Jahren die Vertrautheit mit der Breite des Fachgebietes als wesentliche Voraussetzung für einen zukünftigen Hochschullehrer angesehen [6]. Während sich diese Bedingung in der Untersuchung von 2010 zu 1998 steigerte [5], wurde dies 2021 als etwas weniger wichtig bewertet. Dies mag an dieser Stelle jedoch an der höheren Einschlussrate an Habilitationsordnungen als in den Voruntersuchungen liegen, da die Anzahl derjenigen, die hierauf einen Wert legen, gleichgeblieben ist (*n* = 11).

#### Vorstellung vor der Fakultät (12)

Neun Fakultäten erwarten eine Vorstellung vor der Fakultät oder das Halten eines Vortrages. An 43 % der Fakultäten wird hierfür keine Notwendigkeit gesehen. Zwölf Fakultäten gehen einen Zwischenschritt, in welchem durch die Habilitationskommissionen eine Vorprüfung oder Voranfrage unterschiedlicher Ausgestaltung gefordert wird. Dies stellte für uns die Frage, inwiefern es einer Anmeldung, einer Vereinbarung oder eines verbindlichen Kollegs für die Kandidatinnen und Kandidaten als Anforderung bedarf. Beim überwiegenden Anteil der Fakultäten wird dieses nicht gefordert (26 von 37 = 72 %). Allerdings sind es 3 Fakultäten, welche eine Anmeldung fordern, 7, an welchen eine Vereinbarung oder auch ein Memorandum mit der Betreuerin oder dem Betreuer oder dem Fachmentorat erfolgen muss, und eine Fakultät, in welcher die Einschreibung in ein Programm zur wissenschaftlichen Qualifizierung ähnlich dem der PhD(Philosophical Doctorate)-Programme vorgesehen ist.

## Diskussion

Medizinische Fakultäten und Hochschulen in Deutschland überarbeiten ihre Habilitationsordnungen kontinuierlich, passen diese neuen Gegebenheiten an und halten die Ordnungen somit auf einem aktuellen Stand. In diesem Zusammenhang konnten wir nun mit der dritten Erhebung unter Nutzung des 1998 erstmals erstellten Scoringsmaßstabes [6] feststellen, dass die Gesamtanforderungen erneut signifikant im Vergleich zu 2010 angestiegen sind und dass sich in 11 der 12 untersuchten Scoringkategorien Veränderungen ergeben haben. Einzig unverändert in der Bewertung über 23 Jahre ist die erfolgreich abgeschlossene Promotion als weiterhin überall notwendige Voraussetzung. Neu hinzugekommen ist, dass an 6 Fakultäten eine Promotionsarbeit mit einer Mindestbewertung (z. B. magna cum laude o. Ä.) als Voraussetzung zur Habilitation vorliegen muss. Dies stellt einerseits zwar ein zusätzliches Qualitätskriterium für eine medizinische Habilitation dar, welches andererseits die Voraussetzungen für eine Habilitation jedoch auch weiter nach oben schraubt. Ob diese Voraussetzung geeignet ist, die generelle Zahl der Habilitationen in den Gesundheitswissenschaften in Deutschland wieder ansteigen zu lassen, darf bezweifelt werden. Ferner wird eine akademische Karriere zunehmend weniger langfristig vorausgeplant und auch die Bewertung medizinischer Promotionsarbeiten ist in Deutschland weit weg von einer bundesweit gleichen oder ähnlichen Gütebewertung. Diese Voraussetzung stünde auch einem potenziellen Stellenwechsel für eine Habilitation an einem anderen Ort (national wie international) entgegen, wenn möglicherweise der dringende Wunsch zur Habilitation besteht, jedoch eine schlechtere Promotionsbewertung vorliegt. Im Jahre 1998 konnte noch an 4 Fakultäten eine entsprechend bewertete Promotionsarbeit als schriftliche Habilitationsleistung vorgelegt werden. 2010 war dies noch an einer Fakultät mit dem Prädikat „hervorragend“ möglich und in der aktuellen Untersuchung findet sich diese Möglichkeit in keiner der eingeschlossenen Habilitationsordnungen mehr. Die schriftliche Habilitationsarbeit dient dazu, dass die Bewerberin oder der Bewerber die Bearbeitung eines Problemfeldes durch kontinuierliche Beschäftigung nachweist und dass dies über die nur einfache Dokumentation von Untersuchungen signifikant hinausgeht. Nach heutigem Verständnis ist es in diesem Zusammenhang unverständlich, dass es scheinbar einmal erlaubt war, die wissenschaftliche Tätigkeit für eine Habilitation mit einer hervorragenden Dissertationsleistung zu kompensieren. Die Promotionsarbeit dient im eigentlichen Sinne nur dazu, dass man eine erste über das allgemeine Studienziel hinausgehende Befähigung zu selbständiger wissenschaftlicher Arbeit in einem eng umschriebenen Forschungsumfeld nachweist. Die Habilitationsschrift fordert die Personen jedoch heraus, die erhaltenen wissenschaftlichen Ergebnisse umfassender abzuhandeln und, bei aller Detailliertheit der Ergebnisse, diese in einen globalen Kontext zu setzen. Eine Promotionsschrift kann diesem Anspruch allein vom begrenzten Seitenumfang her schon nicht gerecht werden und so ist es nur nachvollziehbar, dass nun keine Fakultät dies mehr als Habilitationsleistung anerkennt.

Die Bewertung der Habilitationsordnungen mithilfe eines starren Scoringsystems wurde bereits durch die Erstbeschreiber als mögliche zweifelhafte und unfaire Limitation beschrieben. Zusätzlich sind die Ergebnisse auch der hier erneut vorliegenden Bewertung insofern limitiert, als dass nicht alle medizinische Fakultäten eingeschlossen worden sind. Die Aussagen dieser Arbeit betreffen somit jedoch den überwiegenden Anteil medizinischer Fakultäten und Hochschulen in Deutschland. Des Weiteren muss die Aktualität des nun mittlerweile 23 Jahre alten Scoringmaßstabes berücksichtigt werden, der an der ein oder anderen Stelle den Bewertern Grundlage zur Diskussion eröffnet hat. Die Anpassung und Weiterentwicklung des Scoringmaßstabes ist bereits Grundlage fortführender Arbeiten im Rahmen der KARiMED-Studie. Dennoch wurde dieser für diese longitudinale Betrachtung herangezogen. Mitunter haben die Kategorien hierdurch Deckeneffekte, weil nahezu alle Fakultäten dieses Kriterium mit der damals getroffenen höchsten Bepunktung erfüllen, wie dieses in der Kategorie Publikationsleistung in Tab. 2 ersichtlich ist. Zwar erreichen in unserer Bewertung 34 von 37 Ordnungen den Höchstwert, jedoch ist die Ausgestaltung der Publikationen, wie am Zielkriterium selbst erkennbar, äußerst heterogen.

Weineck et al. fanden 2014 in einer Untersuchung zur medizinischen Habilitation im deutschsprachigen Raum heraus, dass die Art und Anzahl der Publikationen das häufigste erwähnte Kriterium (42 %) zur Beurteilung der wissenschaftlichen Qualifikation der Habilitandinnen/ Habilitanden ist, direkt gefolgt von der Autorenposition mit 40 %. Interessanterweise waren Zitationsindizes, obwohl seit langem in der Diskussion, 2014 noch von wenig Interesse in den Habilitationsordnungen [13]. Alawi et al. fanden heraus, dass im Durchschnitt nach der Habilitation insgesamt 11 Veröffentlichungen erforderlich sind (max. 24, min. 6) und hierbei im Durchschnitt 6 Veröffentlichungen mit erster oder letzter Autorenschaft (max. 16, min. 4; [1]). Strauss und Kollegen konnten aktuell berichten, dass eine bestimmte Anzahl an Erst- bzw. Letztautorenschaften in allen Ordnungen zur Habilitation festgeschrieben ist, wobei sich die Anzahl zwischen den Fakultäten deutlich unterscheidet. Zudem sollen die entsprechenden Publikationen in Peer-review-gelisteten Zeitschriften oder Journals mit Impact-Faktor veröffentlicht sein.

Die Publikationswertigkeit wird mit unterschiedlichen Scoringinstrumenten oder Staffelungen in den Habilitationsordnungen beschrieben, bis eine Mindestpunktzahl erreicht wird, mit welcher diese Anforderung als erfüllt angesehen werden kann [10]. Für die Bewertung der publikatorischen Leistung spielen Case-Reports eine nur untergeordnete Rolle. Sie können zwar aufgelistet werden, jedoch fließen sie nicht in die Bewertung der wissenschaftlichen Publikationsleistung mit ein.

Didaktische Erfahrungen sind zunehmend von Bedeutung, insbesondere bei der Erteilung einer Venia Legendi, also der Lehrbefugnis für ein Fachgebiet. Der Stellenwert der Lehre wurde in der Untersuchung von Weber und Kollegen im Jahre 2000 festgestellt. In dieser Untersuchung konnte gezeigt werden, dass die Evaluation der Lehre zum Jahrhundertwechsel an deutschen medizinischen Fakultäten etabliert schien und einige Aktivitäten zur Verbesserung stattfanden. Ein Qualitätsindikator zur Bewertung von Lehrleistungen konnte hingegen nicht erfasst werden [12]. Auch in einer Folgeuntersuchung zur Erteilung der Lehrbefugnis 2005 konnte nur ein heterogenes Bild des Anforderungskatalogs der deutschen medizinischen Hochschullehre beschrieben werden [2]. In beiden Untersuchungen wurde konsekutiv die Einführung messbarer Parameter für durchgeführte Lehrleistungen gefordert. Ob und wie die Lehre an den medizinischen Fakultäten aktuell erfasst wird, stellt sich auch 15 Jahre später weiterhin uneinheitlich dar, jedoch wird der Bereich der Medizindidaktik als zunehmend wichtiger Baustein zur Erlangung einer Habilitation angesehen. Zwar wird die Erlangung spezifischer didaktischer Fähigkeiten mittlerweile fast überall vorausgesetzt, jedoch werden sehr unterschiedliche Bedingungen, wie in der geforderten Mindeststundenanzahl zu erkennen ist, gestellt [10]. Des Weiteren nimmt die personenbezogene Evaluation der Habilitandinnen/Habilitanden durch Studierende oder andere Fakultätsmitglieder weiter zu. Dies kann einerseits als klares Zeichen einer weiteren Aufwertung des Stellenwertes der Lehre in der Medizin angesehen werden. Andererseits sind jedoch weiterhin keine einheitlichen Qualitätsindikatoren zur Lehrevaluation beschrieben, welche einer transparenten Bewertung der Lehrleistung zugrunde liegen könnten.

Die Vorstellung des Scoringsystems im Jahre 1998 diente zum einen der Beschreibung eines einheitlichen Bewertungsmaßstabes, zum anderen sollten jedoch auch Möglichkeiten zur Vereinheitlichung der Habilitationsordnungen aufgezeigt werden. In der aktuellen Bewertung kann hier im Vergleich zu 2010 wieder eine breitere Streuung festgestellt werden, im Sinne einer geringeren Vereinheitlichung der nationalen Habilitationsvoraussetzungen. In Vorstudien der KARiMED Studie stellte eine Vereinheitlichung sowohl der Promotions- als auch der Habilitationsordnungen auf nationaler Ebene ein dringender Reformwunsch von sowohl Medizinstudierenden, akademischem Mittelbau, als auch der beurteilenden Kommissionen dar [7, 8]. Mithilfe dieser nun über 23 Jahre vorliegenden Längsschnittstudie kann diesem Wunsch nicht vollumfänglich entgegengekommen werden. Zwar aktualisieren die Fakultäten ihre Ordnungen von Zeit zu Zeit, jedoch spricht einer bundeseinheitlichen (Rahmen‑)Habilitationsordnung aktuell nicht nur das föderale Bildungssystem in Deutschland entgegen, sondern auch der dringende Wunsch nach der Aufrechterhaltung der wissenschaftlich-akademischen Selbstständigkeit der Universitäten per se. Zwar kann die Habilitation weiter als sehr traditionelle akademische Prüfung angesehen werden, die in Deutschland weiterhin ihre Berechtigung besitzt und zuletzt auch der Bologna-Reform getrotzt hat. Gleichwohl sollte gerade im Hinblick auf den Bologna-Prozess z. B. eine vollständige Anerkennung bereits erfüllter Leistungen an anderen Hochschulorten möglich sein und damit die Möglichkeit für die Kandidatinnen und Kandidaten zu schaffen, ihre Forschungsarbeiten auch an anderen Orten ohne Verluste fortführen zu können. Hierfür sind zwingend einheitliche Ordnungen, nicht nur für die Habilitation, sondern auch für die Promotion, von entscheidender Bedeutung. Neben den Mindestanforderungen für ein nationales Rahmenwerk akademischer Verfahren spielt allerdings die Ausgestaltung des Arbeitsplatzes von Ärztinnen und Ärzten des aktuell akademischen Mittelbaus ebenfalls eine zentrale Rolle. Obwohl an einigen Universitäten akademische Karrierepfade durch Clinician-Scientist- oder Tenure-Track-Programme ermöglicht werden, sind die Voraussetzungen für eine Vereinbarkeit von Familie/Freizeit und Beruf, insbesondere für Frauen, weiterhin schlecht [4]. Eine erfolgreiche Parallelisierung ist nur mit flexiblen Arbeitszeitmodellen, weniger starren Laufbahnstrukturen und adäquater Kinderbetreuung am Dienstort möglich, um auch eine Familie in die Karriereplanung ohne signifikante Einbußen für Männer wie Frauen integrieren zu können.

## Fazit

Zusammenfassend kann also festgestellt werden, dass die Voraussetzungen für eine medizinische Habilitation in Deutschland weiter angestiegen sind. Die detailliertere Beschreibung von Habilitationsvoraussetzungen kann hier als direkter Hinweis auf eine Verbesserung der Transparenz angesehen werden. Durch die nun breitere Streuung der Scoringwerte im Vergleich zur Voruntersuchung zeigt sich hingegen auch, dass ein einheitlicher Bewertungsmaßstab für Deutschland wieder in die Ferne rückt.
